# Nutritional Strategies for the Preservation of Fat Free Mass at High Altitude

**DOI:** 10.3390/nu6020665

**Published:** 2014-02-13

**Authors:** Stacie L. Wing-Gaia

**Affiliations:** Division of Nutrition, University of Utah, 250 South 1850 East, Salt Lake City, UT 84112, USA; E-Mail: stacie.wing@health.utah.edu; Tel.: +1-801-585-9623; Fax: +1-801-585-3874

**Keywords:** hypoxia, mountaineering, body composition, muscle wasting, protein synthesis, leucine

## Abstract

Exposure to extreme altitude presents many physiological challenges. In addition to impaired physical and cognitive function, energy imbalance invariably occurs resulting in weight loss and body composition changes. Weight loss, and in particular, loss of fat free mass, combined with the inherent risks associated with extreme environments presents potential performance, safety, and health risks for those working, recreating, or conducting military operations at extreme altitude. In this review, contributors to muscle wasting at altitude are highlighted with special emphasis on protein turnover. The article will conclude with nutritional strategies that may potentially attenuate loss of fat free mass during high altitude exposure.

## 1. Introduction

It is well-documented that weight maintenance above 5000 m is extremely difficult, if not impossible, in free living adults. A number of explanations have been proposed including anorexia, elevated basal metabolic rate, body water loss, and altered satiety hormones. However, the majority of studies support that altitude-induced weight loss is largely a function of negative energy balance secondary to inadequate energy intake [1,2,3,4,5,6,7,8,9]. Energy expenditure is elevated at altitude and is equivalent to moderate to high intensity exercise conducted at sea level [3]. When this energy expenditure is combined with suboptimal energy intakes of 50%–70% of daily requirements weight loss occurs [1,2,3,4,5,6,7,8,9].

Of particular concern is the composition of weight loss. Above 5000 m fat free mass (FFM) accounts for as much as 60%–70% of weight loss [1,8,9]. Less clear is why FFM comprises such a large percentage of weight loss. Decreased physical inactivity, sleep cycle disruption, cold exposure, and hypoxia and nutrition-related changes in protein metabolism may all play a role in muscle wasting associated with high altitude. Fat free mass is an important participant in the regulation of metabolism, serving as a reservoir of glucogenic amino acids and nitrogen. Loss of FFM compromises aerobic capacity [10], muscle strength [11], physical performance [12], and immune function [13] at high altitude increasing the risk of illness and injury in extreme environments.

Currently, there is limited research on the mechanisms of muscle wasting at altitude. In this review potential contributors to muscle wasting at altitude are discussed with a special emphasis on the independent effects of hypoxia and concurrent altitude-related nutritional issues (*i.e.*, caloric restriction and low protein intake) on protein turnover as influenced by protein synthesis and protein degradation. Nutritional strategies that may potentially attenuate loss of FFM during high altitude exposure are reviewed.

## 2. Weight Loss and Body Composition Changes with Altitude Exposure

Weight loss appears to be an unavoidable consequence of sustained hypobaric hypoxia in both laboratory and field environments [1,2,3,4,5,6,7,8,9]. Several studies have reported body weight losses of 5%–15% [1,5,7,8,14] with losses primarily a function of both the duration of hypoxic exposure and altitude obtained [15,16]. Weight loss can be reduced but not completely abated below 5000 m with carefully prescribed dietary intake and physical activity [17,18,19], but above 5000 m weight maintenance becomes a significant challenge [1,3,4,14]. Although normobaric hypoxia most likely induces weight loss as well, there is scant research comparing normobaric hypoxia and hyopobaric hypoxia on anorexia or specifically energy metabolism. There are some differing influences upon certain respiratory and cardiac functions [20], postural stability [21], oxidative stress [22], and acute mountain sickness (AMS) [23], but in the final analysis, it is the degree of oxygen saturation of hemoglobin and myoglobin that seems to exert the most significant effect upon metabolic processes [20,24].

The reasons for altitude-induced weight loss are multifactorial. At high altitude, appetite and consequently energy intake is greatly reduced. The majority of studies report a 30%–50% decrease in energy intake during high altitude sojourns [1,2,3,5,6,8,9,14]. Following acclimatization at 4300 m appetite generally improves, but most studies support that appetite continues to decrease above 5000 m [15]. Altitude-associated anorexia has been correlated with acute mountain sickness (AMS), a high altitude illness characterized by headache, dyspnea, nausea, vomiting, anorexia, fatigue, and insomnia [25]. However, anorexia may also occur independent of AMS [14]. Satiety hormones are also influenced by hypoxia. Leptin, a key neuroendocrine appetite suppressant, significantly increased following a 17 h overnight normobaric hypoxic exposure simulating 4100 m [26]. Conversely, under hypobaric hypoxic conditions (4300 m), leptin did not change from sea level in either calorically restricted or adequately fed individuals [27]. Further research is warranted to examine the role of the neuroendocrine system in appetite regulation at altitude.

In addition to suboptimal energy intakes, energy expenditure is increased with altitude exposure. This increase is largely attributed to a substantial but transient increase in basal metabolic rate (BMR) [17,24]. Doubly labeled water experiments on Mt. Everest climbers indicate physical activity energy expenditure values of 1.85–3.0 times sea level resting energy expenditure (REE) [2,3,5,17]. To put this in perspective, an REE multiple of 1.5 is equivalent to low/sedentary activity and a REE multiple of five equivalent to participating in the Tour De France [28]. The above values reflect activity typical of moderate activity [3]. In summary, weight loss appears to be more closely related to inadequate energy intake than extreme energy expenditure. The result is a loss of fat mass but also the undesired loss of FFM [5] (Table 1).

### Body Composition Changes

Caloric restriction induces weight loss which is generally a mix of water, fat and FFM. With caloric restriction under normoxic conditions (*i.e.*, sea level), FFM comprises approximately 25% of body weight loss [29]. This can be mitigated through diet (*i.e.*, increased protein) [30,31,32,33] and exercise [29]. However, with caloric restriction under hypoxic conditions (*i.e.*, high altitude) FFM comprises as much as 60%–70% of weight loss [1,9,34]. In subjects exposed to 40 days of hypobaric hypoxia equivalent to 8848 m, FFM comprised 67% of body weight loss with a corresponding 17% reduction in thigh cross-sectional area [1]. In field conditions, Hoppler *et al.* [10] found a similar 20% reduction in the *vastus lateralis* cross-sectional area in 14 mountaineers following eight weeks above 5000 m. Further, on an ascent of Mt. Shishapangma (8046 m), subjects lost 1.9 kg FFM compared to 0.9 kg fat mass [9]. Reynolds *et al.* [5] also reported a reduction in absolute (kg), but not relative (%), FFM in subjects climbing Mt. Everest.

There are some studies that support a greater loss of fat mass (FM) than FFM at high altitude. However, differences may be due to altitude attained and limitations of measurement techniques. Fulco *et al.* [35] compared whole body bioelectrical impedance analysis (BIA) and 6-site skinfold measurements to densitometry (hydrostatic weighing) and found that both BIA and skinfolds overestimated FFM loss compared to densitometry. Tanner *et al.* [7] compared three measures of body composition in five climbers on Mt. McKinley, Alaska with similar results. Fat free mass was found to be the predominant portion of weight loss for both 12-site skinfolds and magnetic resonance imaging (75% and 62%, respectively), but not densitometry (77% FM; 23% FFM). Bioelectrical impedance analysis in particular is an unreliable measure of body composition at high altitude due to fluid perturbations associated with altitude exposure [35]. Further, skinfolds require careful training and tightly controlled hydration and exercise. Measurement of skinfolds with ultrasound is a more reliable method than with calipers [36] and has been used successfully in a high altitude field study [37]. However, body weight is included in skinfold prediction equations calculating FM and FFM and body weight is influenced by body water loss. Dual Energy Xray Absorptiometry (DXA) is generally recognized as the gold standard to measuring body composition, but currently whole body portable DXA scans are not available for field applications at high altitude. Field measurements of body composition are an area of much needed research.

**Table 1:** Body composition changes at altitude.

Reference	*N*	Conditions	Altitude (m)	Days at Altitude	Total Weight Loss (kg)	Body Composition Method	Body Fat Loss (kg)	FFM * Loss (kg)	% Weight Loss as FFM
Boyer and Blume [38]	14 **	Field	<5400 >5400	23 26	1.9 4.0	Skinfolds	1.34 1.2	0.56 2.8	29.5 70.0
Rose *et al.* [1]	8 males	Hypobaric chamber	Up to 8846	38	7.4	Densitometry	2.51	5.05	66.8
Fulco *et al*. [35]	16 males	Field	3700–4300	16	5.9	Densitometry Skinfolds Bioelectrical impedance	3.46 2.53 1.34	2.44 3.37 4.56	41.4 57.1 77.3
Westerterp *et al.* [2]	3 male 2 female	Field	5300–8872	30 ^†^	2.2	Skinfolds	1.4	0.8	36.4
Westerterp *et al.* [3]	4 male 2 female	Field	6542	21	4.9	Skinfolds	3.5	1.3	27.0
Pulfrey and Jones [9]	5 male 1 female	Field	5900–8046	40 ^†^	3.7	Skinfolds	0.9	1.9	51.4
Armellini *et al.* [8]	10 male 2 female	Field	≥4500	16	3.3	Bioelectrical impedance	2.2	1.1	33.3
Tanner *et al.* [7]	5 male	Field	2200–4300	21	4.2	Densitometry Skinfolds Magnetic resonance	3.2 1.1 1.7	1.0 3.2 2.5	23.0 75.0 62.0
Wing-Gaia *et al.* [37] ^‡^	10 male 8 female	Field	2835–5364	13	Control: 1.9 Leucine: 1.8	Ultrasound skinfolds	0.6 1.1	1.2 0.8	66.0 42.0

* Fat free mass; ** Gender not specified; ^†^ Study period was day 17–25 [2] and day 30–38 [9]; ^‡^ Supplemented with leucine (7 g/day) [37].

## 3. Potential Mechanisms of Altitude-Induced Muscle Wasting

**Figure 1:**
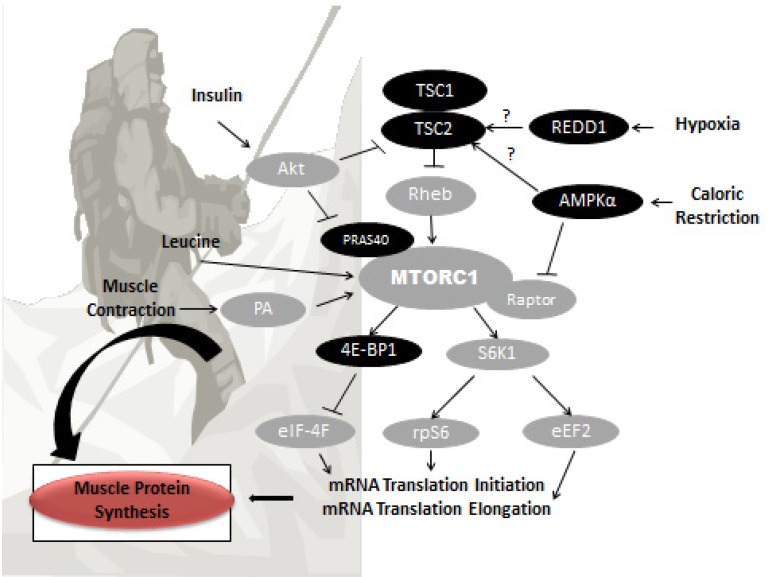
A simplified schematic diagram of the mammalian target of rapamycin complex 1 (mTORC1) signaling pathway and proposed cellular regulation of muscle protein synthesis in response to hypoxia, caloric restriction, insulin, muscle contraction, and leucine. Proteins labeled as gray are positive regulators of mTORC1 and/or muscle protein synthesis and proteins labeled black are negative regulators of mTORC1 and/or muscle protein synthesis. TSC1, tuberous sclerosis complex 1; TSC2, tuberous sclerosis complex 2; Rheb, Ras-homologue enriched in brain; REDD1, gene regulated in DNA damage responses and development; AMPKα, AMP-activated protein kinase alpha; PRAS40, proline-rich Akt substrate 40; Raptor, regulatory associated protein of mTOR; S6K1, p70 ribosomal S6 kinase 1; rpS6, ribosomal protein S6; eEF2, eukaryotic elongation factor 2; 4E-BP1, 4E binding protein 1; eIF-4F, eukaryotic initiation factor 4F; PA, phosphatidic acid; Akt, protein kinase B.

Muscle wasting is a well-documented occurrence with chronic high altitude exposure. Skeletal muscle is of particular importance given it contains the majority of body proteins and comprises almost half of human body weight [39]. As such skeletal muscle accounts for a large proportion of whole body protein turnover. Protein turnover is a dynamic process with approximately 1%–2% of skeletal muscle broken down and synthesized daily under normal ambient conditions [40]. A chronic imbalance of proteins synthesized or degraded (or both) will ultimately influence the size of muscle mass. It is conceivable that altitude could exert its influence upon muscle wasting (turnover) by altering muscle protein synthesis or degradation. Control of breakdown is largely regulated through the ubiquitin proteasome (UP), lysosomal, and calpains systems [41]. Muscle contraction and branched-chain amino acids are potent stimulators of muscle protein synthesis (MPS) and are important daily anabolic stimuli necessary to maintain muscle mass. From a cellular perspective, the mammalian target of rapamycin (mTOR) pathway is a central point of control for muscle protein synthesis and occupies a central point of convergence for nutritional and contractile anabolic signals. It is therefore a useful point of reference in discussing mechanisms that may contribute to muscle loss at altitude (Figure 1).

### 3.1. Hypoxia

Perhaps the largest contributor to muscle wasting at altitude is hypoxia. Evidence suggests that hypoxic exposure may impair MPS through downregulation of mTOR by the hypoxia-induced REDD1 gene [42]. Of particular importance is that this downregulation is independent of hypophagia [43] suggesting that while nutrition may be able to mitigate muscle wasting, it most likely will not completely prevent muscle wasting. Early studies in rat models noted decreased MPS following 6 h of hypobaric hypoxia [44] and impaired growth rate following 14 days of high altitude exposure [45]. Bigard *et al*. [46] also found depressed growth rate in rats exposed to hypobaric hypoxia (6000 m) for 26 days despite high dietary protein intake. In humans, Vigano *et al*. [47] found a reduction in mTOR expression following 7–9 days of hypobaric hypoxia (4559 m). Conversely, Etheridge *et al.* [48] exposed seven healthy young adult men to 3.5 h of normobaric hypoxia and found no effect on MPS at rest. However, when these participants completed a bout of resistance exercise following hypoxic exposure, the normal contraction-induced increase in MPS was significantly blunted (1.4 *vs.* three-fold increase in MPS). Similarly, in a comparison of subjects who were either flown or walked to 4559 m, those walking had a 35% increase in fractional synthetic rate compared to those not walking [49]. However, the increase in MPS following walking was much lower than comparable sea level exercise studies [50] suggesting that post-exercise MPS was blunted in some manner by acute hypoxia.

Limited research suggests that muscle proteolysis may also be affected with hypoxic exposure. Using a rat model, Chaudhary *et al*. [51] examined the effects of 3, 7, and 14 days of hypobaric hypoxia (7620 m) on protein turnover under eucaloric conditions. Following physical activity, there was a 1.5-fold increase in protein synthesis. However, there was a five-fold increase in protein degradation secondary to upregulation of the UP and calpains systems. In humans, Holm *et al*. [52] reported similar findings. Following 7–9 days of acclimatization at 4559 m, whole body protein degradation was increased. In summary, limited data suggests MPS is downregulated and muscle proteolysis upregulated with hypoxia. Downregulation of MPS can partially be overcome with resistance exercise, but this may not be feasible at altitude where strenuous physical exercise is often an ongoing daily occurrence. Although it is important to note that hypoxia-induced downregulation of MPS occurs regardless of nutritional status [43,46], nutritional deficits (*i.e.*, calories and protein) may potentiate the problem. Therefore, targeting diet and nutrient intake may be a more practical approach to influencing MPS at altitude.

### 3.2. Caloric Restriction and Suboptimal Protein Intake

As previously discussed, a primary contributor to weight loss at altitude is low energy intake. Energy status influences MPS. Thus it is likely subcaloric intake contributes to both impaired MPS and increased proteolysis at altitude. This effect is exacerbated in combination with hypoxia as demonstrated by Favier *et al.* [43] who exposed rats to hypobaric hypoxia for 26 days. A 210% increase in REDD1 protein level was noted in hypophagic rats compared to pair-fed normoxic rats. However, a 370% increase in REDD1 was noted in hypophagic rats compared to ad libitum fed normoxic rats [43]. Sea level studies indicate acute caloric restriction (less than three weeks) activates AMP-activated protein kinase (AMPKα), an inhibitor of mTOR [53]. Conversely, in a study evaluating the effects of 10 days of 20% energy deficit with 1.5 g protein/kg/day, AMPKα was not altered [54]. However, reduced phosphorylation of intracellular signaling proteins Akt and eukaryotic initiation factor 4E binding protein 1 (4E-BP1) resulted in a 19% decrease in MPS with energy deficit compared to weight maintenance [54]. In the absence of sufficient energy, whole body proteolysis is also increased as amino acids are mobilized and oxidized for energy or gluconeogenesis. Although the mechanism(s) remain to be fully elucidated, Carbone *et al.* [55] demonstrated that 21 days of 40% caloric restriction upregulated gene expression of the UP system active in muscle proteolysis. In summary, acute caloric restriction results in decreased protein synthesis and increased proteolysis. It is unknown, but likely, that caloric restriction associated with high altitude exposure elicits a similar response in protein turnover.

As a consequence of low energy intake, protein intake is also compromised with high altitude exposure. Protein intake, particularly the branched-chain amino acids (BCAA), is critical for the regulation of MPS [56]. Several sea level studies have examined the effects of varying protein prescriptions on retention of FFM during energy deficits induced by hypocaloric diets and exercise. In a study of twenty resistance trained men, 2.3 g protein/kg/day was superior (20% greater) than 0.9 g/kg for retention of FFM following a 40% caloric restriction for two weeks [32]. Pasiakos *et al.* [31] found no benefit of protein in excess of twice the Recommended Dietary Allowance (RDA) (1.6 g protein/kg) when comparing the RDA (0.8 g/kg), 2 × RDA (1.6 g/kg), and 3 × RDA (2.4 g/kg) on FFM retention following 40% caloric restriction for 21 days. Using an exercise-induced energy deficit of 1000 kcal/day for seven days, Pikosky *et al*. [33] similarly found that 1.8 g protein/kg was superior to 0.9 g/kg for maintenance of nitrogen balance. This is in accordance with a meta-analysis that found protein intakes of 1.05–1.20 g/kg associated with a 0.78 kg additional FFM retention and amounts >1.2 g/kg associated with 0.96 kg additional FFM retention during caloric restriction [30].

Regarding high altitude protein supplementation studies, data is limited. In rats supplemented with 10, 20 or 40 g of protein for 26 days at 6000 m, protein supplementation did not prevent the decrease in muscle growth [46]. In ski mountaineers supplemented with 1.5 g or 2.5 g casein protein/day for seven days at 2500–3800 m, body mass did not change. However, 50% maximal voluntary contraction endurance of the quadriceps muscle post-skiing was decreased with the high protein dose [57]. In summary, under normoxic conditions when in energy balance, evidence suggests increasing protein intake has no further advantage on increasing FFM. However, during caloric restriction, increasing protein intake to approximately twice the RDA (1.6 g/kg) improves nitrogen balance. Whether this level of protein intake is possible to achieve or even beneficial during prolonged high altitude exposure under hypocaloric conditions is undetermined.

## 4. Nutritional Strategies for Retention of FFM at Altitude

Targeting two primary areas of nutrition may be useful to improve FFM retention at altitude: caloric intake and protein intake. Of these two areas, only the latter would seem practical and feasible. As demonstrated in previous studies, weight maintenance is extremely difficult at progressively higher altitudes above 5000 m. Butterfield *et al.* [17] was able to mitigate weight loss in four of seven subjects at 4300 m by providing food matched precisely to energy expenditure. Kayser *et al.* [18] was able to minimize weight loss at 5050 m by limiting exercise to minimal tasks and providing palatable food in a comfortable environment. However, field studies at higher elevations were unable to replicate these findings. Macdonald *et al.* [58] found that neither additional calories (+11.2 ± 4.5 kcal/kg/day body weight) nor higher initial body fat mass attenuated loss of FFM during a 21-day Himalayan trek. Reynolds *et al.* [6] provided highly palatable food to Everest base camp personnel and climbers. Despite palatable foods, food intake decreased dramatically and weight (and FFM) loss occurred. There have been anecdotal accounts and author observations of weight maintenance in climbers who force themselves to continually eat, but for most this is extremely difficult. Therefore, increasing caloric intake may not be the most useful strategy for retention of FFM at altitude. However, manipulating protein intake, more specifically type and amount of protein, may prove a more practical strategy.

### 4.1. Protein and FFM Retention

Given the present body of research, increasing protein intake at altitude would appear a logical choice to aid retention of FFM. However, there are many limitations to this approach. At altitude increasing protein intake may not be feasible or practical. In a recent trek to Everest base camp, trekkers supplemented with protein were able to maintain a protein intake of only 1.1 g protein/kg/day [37] which is below the 1.6 g protein/kg/day needed to support FFM retention during negative energy balance [31]. Further, protein is thermogenic with an energy cost of approximately 20%–30% [59]. In an environment where oxygen is limited, increasing protein intake may be too “energetically expensive”. In addition, at extreme altitudes it is unknown just how much protein can be tolerated. Early reports proposed malabsorption as an issue at extreme altitude [38]. Although largely disproven [3,60], there may be some individuality regarding the amount of protein that can be ingested during extreme altitude expeditions. Lastly, perhaps the most problematic issue with protein is its satiating effect [61]. Increased satiety is considered a favorable consequence of high protein intake for sea level weight loss (dieting). However, adding more protein could potentiate the problem of blunted appetite at altitude [62] and further deplete glycogen levels and impair performance, particularly if protein consumption reduces carbohydrate intake [46,57]. Currently, no research has been conducted evaluating the effects of protein supplementation on satiety at altitude. Therefore, the best approach may be to stimulate MPS and/or decrease proteolysis with a low volume protein supplement high in MPS-promoting branched-chain amino acids and, in particular, leucine. It is worthy to note that leucine metabolites such as β-hydroxy-β-methylbutyrate (HMB) may also promote muscle anabolism, but to a lesser extent than leucine and via different or additional mechanisms to leucine [63]. Although potentially beneficial, there is currently no data evaluating the effects of HMB on muscle wasting at altitude. Therefore, the remaining discussion will focus on the direct cell signaling nutrient leucine and its role in MPS.

### 4.2. Leucine and FFM Retention

Leucine is a branched-chain amino acid that is not only a substrate for the synthesis of new proteins but is critical in mTOR cell signaling and muscle protein synthesis regulation [64,65,66]. Leucine may also help regulate proteolysis through downregulation of the UP pathway [67] and thereby has been marketed as both an anabolic and anticatabolic nutrient. Recent investigations have demonstrated that leucine in dietary protein is the main determinant of postprandial MPS [68] with liquid sources being superior to solid sources in inducing peak leucine levels [69]. Previously, leucine has been manipulated to increase muscle mass in athletes and FFM retention during hypocaloric diets at sea level (see reviews by [70,71]). More recently, due to its regulatory function on skeletal muscle, leucine has been manipulated to improve FFM retention in muscle wasting conditions associated with cancer [72] and aging [56].

The above information presents the possibility of a unique application of leucine to muscle wasting at altitude. Metabolite profiling in yeast cells has shown dramatically low levels of cellular leucine secondary to downregulation of amino acid permeases (leucine transporters) under hypoxic conditions [73]. Further, certain conditions such as endurance exercise and depleted glycogen, both conditions associated with high altitude exposure, increase leucine oxidation [74]. Observations such as these suggest that the hypoxia of altitude may exert similar effects upon leucine transport and oxidation, resulting in a “drain” on leucine availability to participate in protein synthesis. Unfortunately, palatability of amino acids, and in particular, leucine is low. However, if palatability can be achieved, providing supplemental leucine may be a feasible way of retaining FFM without the need to ingest large amounts of protein.

Under normoxic situations where high quality meal protein and energy intakes are sufficient, supplemental leucine would not be expected to exert a stimulatory effect upon protein synthesis. Glynn *et al.* [75] demonstrated that 10 g of an essential amino acid (EAA) mixture typical of a high quality protein (18% leucine) compared to 10 g of EAA mixture enriched with leucine (35% leucine) elicited similar responses in 1-h postprandial MPS despite improved mTOR signaling with higher leucine content. Similarly, in another study 16.6 g whey protein enriched with 3.4 g leucine produced a MPS response equivalent to 20 g whey following resistance exercise [76]. It is when high quality protein intake is insufficient, as commonly experienced with high altitude exposure, that leucine most likely may play a key role in stimulating MPS and FFM retention.

The majority of research supports the amount of 20–25 g of high quality protein ingested at one time as the maximal dose for subsequent MPS stimulation. In situations where it is not possible to ingest sufficient protein (*i.e.*, altitude), or when the muscle is resistant to stimuli (*i.e.*, aging), small amounts of protein enriched with leucine may enhance the MPS feeding response. In a study investigating the effects of 25 g whey protein (3 g leucine) compared to 6.25 g whey protein enriched with a leucine content equivalent to 25 g of whey (0.75 g leucine + 2.25 g leucine) and 6.25 g whey protein (0.75 g leucine), both whey and low dose whey + leucine increased MPS similarly 1–3 h post-feeding. However, the higher dose whey protein was more effective at sustaining increased MPS following resistance-exercise [77]. It is important to note that these results were shown in young, active males. In aging populations, higher protein (*i.e.*, higher leucine) (40 g whey) was found to induce the greatest stimulus in MPS following resistance exercise [78]. Given the similarity between the aging and hypoxia model on blunted post-exercise MPS, leucine may provide the stimulus needed for FFM retention when protein intake is insufficient.

Sea level studies suggest leucine may also be of benefit when ingested during exercise. Although studies have investigated the effects of leucine ingested post-exercise, few have examined the effects of leucine during exercise. Pasiakos *et al.* [79] demonstrated that 10 g EAA (3.5 g leucine) ingested during 60 min of steady state (60% VO_2peak_) endurance exercise increased MPS by 33% compared with 10 g EAA (1.87 g leucine) although both increased Akt and mTOR phosphorylation 30 min post-exercise. Coffey *et al.* [80] reported similar findings examining the effects of leucine on high intensity exercise. In this study, subjects ingested 24 g whey protein, 4.8 g leucine and 50 g maltodextrin prior to two sprint cycling sessions. Myofibrillar protein synthesis was 48% greater than the noncaloric placebo 15 min post-exercise with an associated increase in Akt and mTOR phosphorylation. Although the study design of a noncaloric control limits the applicability of leucine, it does support the benefit of consuming small amounts of protein with leucine and carbohydrate during high intensity exercise that may be experienced at high altitudes.

Three studies have examined the effects of branched chain amino acids (BCAA) and leucine on body composition at altitude. Bigard *et al.* [81] examined the effects of BCAA (7.8 g leucine, 3.4 g isoleucine, 11.2 g valine; 1.44 g protein/kg) compared to carbohydrate supplementation on body composition following six days of ski mountaineering at 2500–3800 m. Body composition and muscular performance were unaffected by BCAA. However, significant weight loss only occurred in the carbohydrate supplemented group (−1.55 kg *vs.* −0.8 kg). In addition, Schena *et al*. [82] investigated the effects of BCAA (5.76 g leucine, 2.88 g isoleucine, 2.88 g valine) on body composition during a 21-day trek at a mean altitude of 3255 m. An increase in lean muscle mass (+1.5%) was noted in the supplemented group with no change in the control group. Because the placebo was noncaloric it is difficult to determine if the positive effects on body composition were due to BCAA or simply from increased energy intake and/or protein. More recently, in double blind randomized fashion supplemental leucine (14 g protein; 7 g leucine) or an isocaloric, isonitrogenous control (11 g protein; 0.34 leucine) was administered twice daily in a hot chocolate beverage during a 13 day trek to Everest base camp. All subjects lost significant body weight. However, FFM comprised 66% of the control weight loss compared with 42% of the leucine weight loss [37]. The altitude attained and duration of altitude exposure limited the extent of weight and FFM loss, but the study did demonstrate that leucine was palatable and well-tolerated up to approximately 5300 m. Further, it is of particular importance that there was no difference in carefully documented caloric intake between leucine supplemented and nonsupplemented subjects suggesting leucine did not potentiate the increased satiety induced by altitude. More research is needed to determine if leucine is an effective strategy for FFM retention at altitude.

## 5. Conclusions

Chronic high altitude exposure is associated with significant weight loss primarily comprised of FFM. Loss of FFM has negative consequences related to decreased physical performance and increased risk of illness and injury. Studies have demonstrated at lower elevations that when subjects are provided with sufficient food and perform limited activity, energy balance can be maintained. As altitude progresses, weight maintenance becomes virtually impossible. Hypoxia, negative energy balance, and insufficient high quality protein limit the body’s ability to synthesize protein secondary to inhibition of the mTOR pathway and perhaps accelerated proteolysis via upregulation of the UP system. Although this could be viewed as a favorable adaptation in the context of survivability, it is not in terms of performance and health. Increasing caloric and protein intake is difficult at high altitude due to feasibility and perturbations in appetite regulation. Therefore, the most practical strategy to improve FFM retention at altitude may be in the form of supplemental leucine. Clearly more research is needed in this area to determine the exact mechanisms related to altitude-induced muscle wasting, protein requirements and effectiveness of leucine for the retention of FFM during both acute and long-term high altitude exposure. 
